# Revealing the inhibitory effect of VASH1 on ovarian cancer from multiple perspectives

**DOI:** 10.1080/15384047.2023.2285817

**Published:** 2023-11-27

**Authors:** Yan Li, Liang Meng, Ge Lou

**Affiliations:** a Department of Gynecology, Harbin Medical University Cancer Hospital, Harbin, Heilongjiang, China; b Department of Obstetrics and Gynecology, First Affiliated Hospital of Harbin Medical University, Harbin, Heilongjiang, China; c State Key Laboratory of Veterinary Biotechnology, Harbin Veterinary Research Institute, Chinese Academy of Agricultural Sciences, Harbin, China

**Keywords:** *VASH1*, ovarian cancer, suppressor, tumor purity, immune cell infiltration

## Abstract

The function of *Vasohibin-1* (*VASH1*) in human cancer has not been thoroughly or comprehensively examined. Here, we identified the tumor suppressor part of *VASH1* across cancers, including epithelial ovarian tumors. Our study carefully contrasted the expression of VASH1 in pancancer and nontumorous tissues in a public database to explore its regulatory role in clinical prognosis, diagnosis, tumor purity, and immune cell infiltration. Next, we explored the antitumor mechanism of *VASH1* through drug sensitivity, functional enrichment, and phenotypic experiments in ovarian cancer. Research suggests that the expression of *VASH1* in neoplastic tissues is lower than that in normal tissues. *VASH1* affects the OS and RFS of several tumor types. In addition, *VASH1* expression resulted in a high OS and RFS in the diagnosis of tumor and nontumor tissues and negatively regulated tumor purity. Moreover, *VASH1* controls the tumor microenvironment by regulating immunocyte infiltration. In ovarian cancer, *VASH1* can serve as a biomarker to estimate the efficacy of chemotherapy. Functional enrichment analysis suggests that *VASH1* plays a tumor suppressor role by regulating the extracellular matrix receptor pathway. *VASH1* inhibition of the malignant phenotype of ovarian cancer cells was further confirmed by *in vivo* experiments. These results indicate that *VASH1* acts as a cancer-inhibiting factor and potential therapeutic target in ovarian cancer.

## Introduction

Ovarian cancer is a major cause of death in patients with gynecological malignancies. Epithelial ovarian cancer is the most common type of cancer and has a very low five-year survival rate.^1^ Although rapid progress has been made in tumor cytoreductive surgery, cisplatin-based chemotherapy, targeted drugs, and immunotherapy, the clinical results in ovarian cancer patients remain unsatisfactory because most patients have local progression and extensive distant metastases when they are diagnosed.^2^ Although most ovarian cancer patients respond to treatment at the beginning, nearly three-quarters of patients relapse within three years.^3^

Watanabe et al. first reported and named *VASH1* in 2004.^4^ *VASH1* is a member of the angiostatin family of proteins. Inhibition of tumor angiogenesis has been shown to be an effective therapeutic target for cancer patients. To date, diverse proangiogenic factors and angiogenesis inhibitors have been used clinically.^5^ Research has confirmed that endothelial cells express *VASH1*, which can inhibit migration, proliferation, and angiogenesis by controlling the biological function of endothelial cells.^6–8^ *VASH1* is not limited to endothelial cells; at the same time, it is also expressed in human tumor cells.^9,10^ Research has shown that *VASH1* plays a major role in a variety of malignant tumors, including prostate cancer, upper urinary tract urothelial carcinomas, lung cancer, and cervical tumors.^11–16^ Studies have shown that *VASH1* exerts antitumor effects by suppressing vascularization in the TME.^17,18^

Nevertheless, the role of *VASH1* in human pancancer has not yet been comprehensively investigated. Therefore, in our research, we first analyzed the distinct expression of *VASH1* in different tumors and then explored the predictive value of *VASH1* in clinical prognosis and diagnosis. We also studied the effects of *VASH1* on tumor purity and immune cell infiltration. In an effort to further probe the relationship between *VASH1* expression and biological significance, the research finally focused on ovarian cancer, exploring drug sensitivity, functional enrichment analysis, and regulation of the malignant phenotype. The outcome of this study implied the antitumor effect of *VASH1* in pancancer, confirmed by bioinformatics analysis combining experiments, and provided evidence for new targets to inhibit the progression of ovarian cancer.

## Materials & methods

### Gepia

Gene Expression Profiling Interactive Analysis (http://gepia.cancer-pku.cn/) is a communal database for normal and tumor gene expression profiling, including RNA sequencing expression data of over 9700 tumor specimens and over 8500 normal samples. *VASH1* expression levels in pancancer and nontumorous tissues were compared.

### TNMplot database

The TNMplot database (http://www.tnmplot.com) is a online tool for gene expression level in normal, metastatic, and tumor tissues. We used this method to explore the expression level of *VASH1* in diverse tumor and normal tissues.

### TIMER database

TIMER (https://cistrome.shiny apps.io/timer/) utilizes RNA-seq expression profile data to detect the infiltration degree of multiple immune cells in neoplastic tissues. The DiffExp module allowed users to study the differential expression of *VASH1* in all TCGA tumors and adjacent normal tissues.

### Kaplan–Meier Plotter

The K–M Plotter (http://kmplot.com/analysis/) is a survival analysis website with complete and authoritative data, which was used to analyze overall survival and relapse-free survival.

### LinkedOmics

We examined the link between *VASH1* expression and the purity of multiple tumors using the LinkedOmics website (http://www.linkedomics.org/login.php). This website was also used to perform GO and KEGG analyses of genes related to *VASH1* expression in ovarian cancer.

### ROC plotter

The study used ROC plotter (http://www.rocplot.org) to probe *VASH1* expression and drug response using transcriptome-level data from patients with breast, ovarian, and colorectal cancer, and glioblastoma.

### Cell lines and cell culture

All ovarian cancer cell lines (A2780 RRID:CVCL_0134, Caov-3 RRID: CVCL_0203, OVCAR-3 RRID: CVCL_0465, and SK-OV-3 RRID: CVCL_0532) and normal epithelial cell lines (IOSE80, RRID: CVCL_5546) were purchased from Procell Life Technology Co., Ltd. (Wuhan, China) with supporting cell line authentication. The cells were cultured in RPMI 1640 medium (Gibco Company, New York, USA) containing ten percent fetal bovine serum, 100 µg/mL streptomycin, and 100 IU/mL penicillin in a 37°C incubator with 5% CO_2_.

### Cell transfection

Prior to transfection, the cells were grown to cover 60% of the culture flasks. Each flask was cultured with approximately 5 ml serum-free medium, 5 μl Polyplus-Transfection ® (JetPRIME, USA), and 100 pmol pcDNA-*VASH1* (General biol, China) or si-*VASH1* (General biol, China). After 8 h, the medium was replaced with complete medium.

### Quantitative real-time PCR

RT‒qPCR was performed to analyze *VASH1* expression in the samples. RNA was purified using the Total RNA Kit (Omega), and a total of 1,000 μg RNA was reverse transcribed into cDNA using the RT‒PCR Transcriptor First Strand cDNA Synthesis Kit (Roche). qPCR was performed to detect the mRNA expression level using FastStart Universal SYBR Green Master (ROX) (Roche) with the Applied Biosystems StepOnePlus™ Real-Time PCR system (Thermo Fisher Scientific, Inc., USA). Human β-actin was used as an internal reference to determine mRNA expression. The expression levels were calculated using the 2‑ΔΔCq method.

### Western blot analysis

Integral proteins were collected using RIPA buffer (BiYunTian, China). A bicinchoninic acid (BCA) kit (Biotech, China) was used to determine protein concentrations. A total of 20 μg of protein was separated using 12.5% SDS‒PAGE and transferred onto a PVDF membrane (Bio-Rad). After blocking with five percent skim milk for one hour at ambient temperature, the membrane was subsequently incubated in a solution of diluted rabbit polyclonal antibody to *VASH1* (ABclonal, A6148, 1:1000).

### Proliferation assay

A total of 3 × 10^3^ cells were cultivated in 96-well plates, and the culture solution was replaced with fresh RPMI 1640 every day. The Cell Counting Kit-8 (BiYunTian, China) was used to test cell proliferation after 24, 48, and 72 h of incubation.

### Colony formation assay

The cells were seeded into a 6-well plate at a density of 500 cells/well. At 37°C and 5% CO_2_, the cells were cultured for 2 weeks, and the solution was replaced with fresh RPMI1640 every 2 days. The culture was terminated when visible colonies were terminated. The cells were then washed with PBS and fixed with 4% paraformaldehyde for 15 min at room temperature. Finally, the cells were stained with 0.1% crystal violet (Solarbio, China) at room temperature for 20 min, and the staining solution was removed by washing. The colonies were photographed using a digital camera.

### Wounding healing assay

Cells were seeded in 6-well plates and cultured until they covered the monolayer. A (yellow) pipette tip was used to draw a straight scratch to form a wound. Healing was observed under a microscope at 12 and 24 h, and a series of photographs were taken to document the process.

### Statistical analysis

Each experiment was repeated independently at least three times. The statistical analysis in this study was carried out automatically using an online database. *P* values were calculated using an unpaired Student’s t test. *P* value < 0.05.

## Results

### *Variant expression of* VASH1 *in human pancancer*

**Figure 1.**
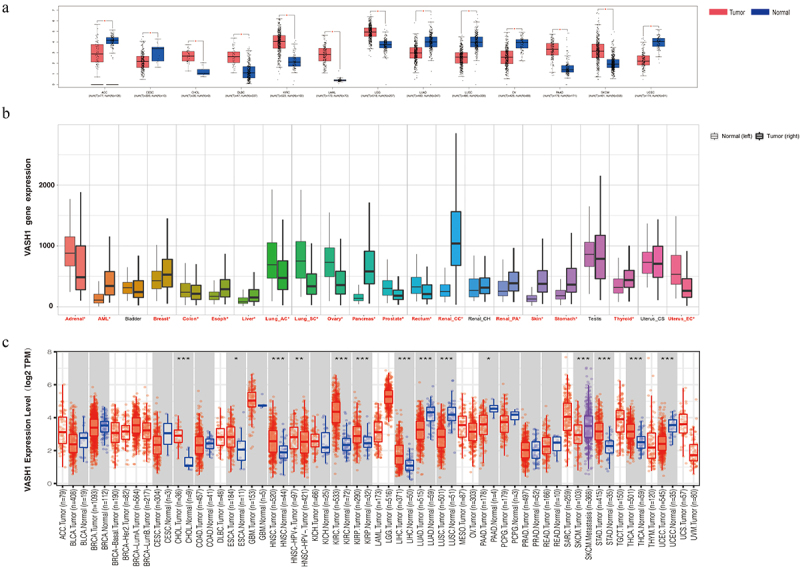
*VASH1* expression level in human pan‐cancer and normal tissue. (a) GEPIA database, (b) TNMplot database, (c) TIMER database.

We explored the differential expression of *VASH1* in distinct carcinomas by using multiple databases. Comprehensive analysis of the GEPIA, TNMplot, and TIMER databases revealed that *VASH1* expression levels were lower in ACC, LUAD, LUSC, OV, and UCEC than in normal tissues. The expression of *VASH1* was higher in CHOL, ESCA, KIRC, KIRP, LIHC, SKCM, and THCA (Figure 1). The results revealed an opposite trend for *VASH1* in different types of tumors.

### *Effect of* VASH1 *expression on clinical prognosis*

**Figure 2.**
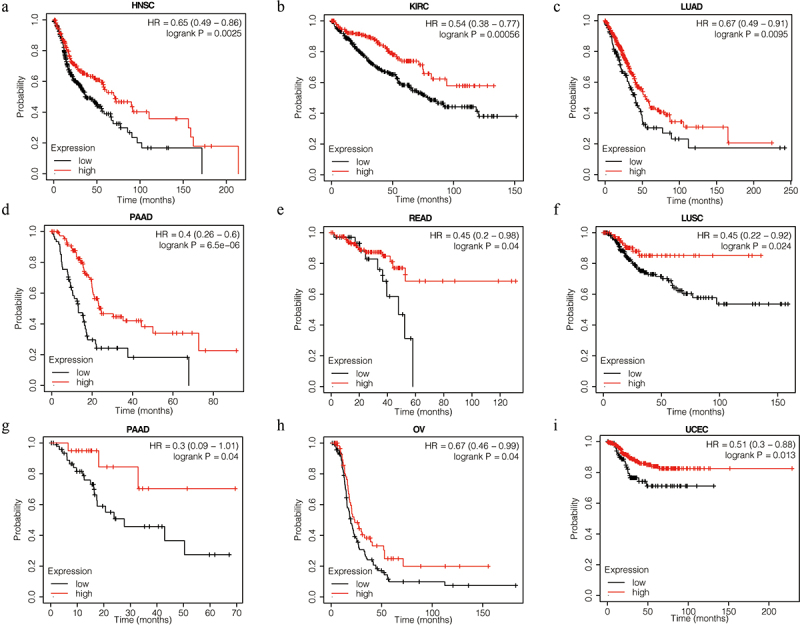
*VASH1* expression is associated with OS and RFS. High expression of *VASH1* correlates with better OS in HNSC **(a)**, KIRC **(b)**, LUAD **(c)**, PAAD **(d)**, and READ **(e)**. High expression of *VASH1* correlates with better RFS in LUSC **(f)**, PAAD **(g)**, OV **(h)**, and UCEC **(i)**. OS, overall survival; RFS, relapse-free survival; HNCS, head and neck squamous cell carcinoma; KIRC, kidney renal clear cell carcinoma; LUAD, lung adenocarcinoma; LUSC, lung squamous cell carcinoma; PAAD, pancreatic adenocarcinoma; READ, rectum adenocarcinoma; OV, ovarian serous cystadenocarcinoma; UCEC, uterine corpus endometrial carcinoma.

K-M plotter was used to predict the clinical prognosis associated with *VASH1*. As shown in Figure 2–e, patients with a high level of *VASH1* mRNA had better overall survival in HNCS (HR = 0.65, *P* = 2.5e-03), KIRC (HR = 0.54, *P* = 5.6e-04), LUAD (HR = 0.67, *P* = 9.5e-03), PAAD (HR = 0.4, *P* = 6.5e-06), and READ (HR = 0.45, *P* = 4.0e-02). Similarly, patients with high levels of *VASH1* mRNA had better RFS outcomes in LUSC (HR = 0.45, *P* = 2.4e-02), PAAD (HR = 0.30, *P* = 4.0e-02), OV (HR = 0.67, *P* = 4.0e-02), and UCEC (HR = 0.51, *P* = 1.3e-02) (Figure 2f–i).

**Figure 3.**
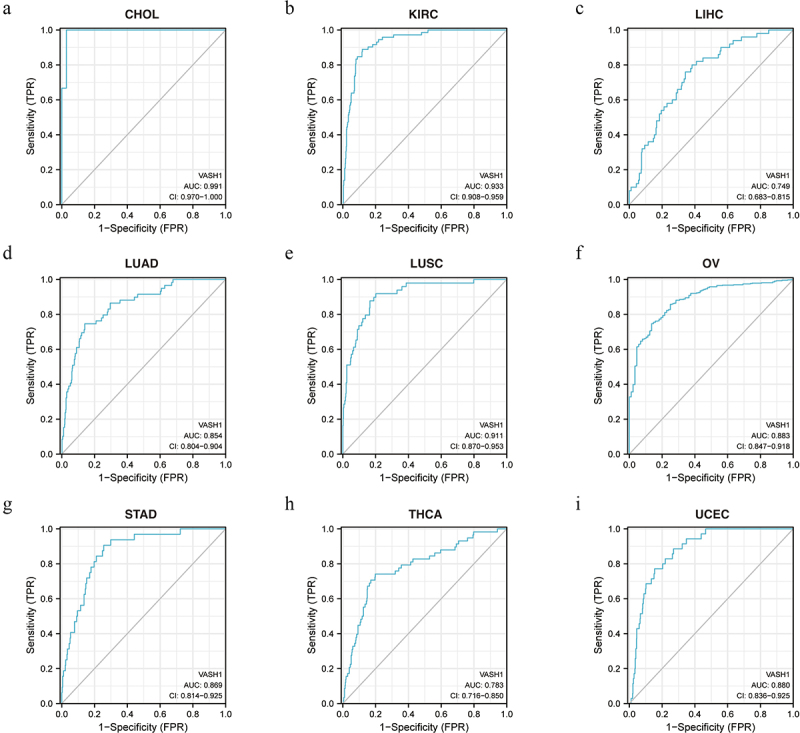
The function of *VASH1* in clinical diagnosis. *VASH1* could be used as a beneficial biomarker for clinical diagnosis in CHOL (a), KIRC (b), LIHC (c), LUAD (d), LUSC (e), OV (f), STAD (g), THCA (h) and UCEC (i). CHOL, cholangiocarcinoma; KIRC, renal clear cell carcinoma; LIHC, liver hepatocellular carcinoma; LUAD, lung adenocarcinoma; LUSC, lung squamous cell carcinoma; OV, ovarian serous cystadenocarcinoma; STAD, stomach adenocarcinoma; THCA, thyroid carcinoma; UCEC, uterine corpus endometrial carcinoma.

To further investigate the function of *VASH1* in clinical diagnosis, we generated ROC curves for different tumors. The results showed that it could be used as a beneficial biomarker for the clinical diagnosis of CHOL (AUC = 0.991, CI = 0.970–1.000), KIRC (AUC = 0.933, CI = 0.908–0.959), LIHC (AUC = 0.749, CI = 0.683–0.815), LUAD (AUC = 0.854, CI = 0.804–0.904), LUSC (AUC = 0.911, CI = 0.870–0.953), OV (AUC = 0.883, CI = 0.847–0.918), STAD (AUC = 0.869, CI = 0.814–0.925), THCA (AUC = 0.783, CI = 0.716–0.850), and UCEC (AUC = 0.880, CI = 0.836–0.925) (Figure 3). These findings indicated that *VASH1* is a valuable biomarker for clinical guidance.

### VASH1 *negatively affected the purity of tumors*

**Figure 4.**
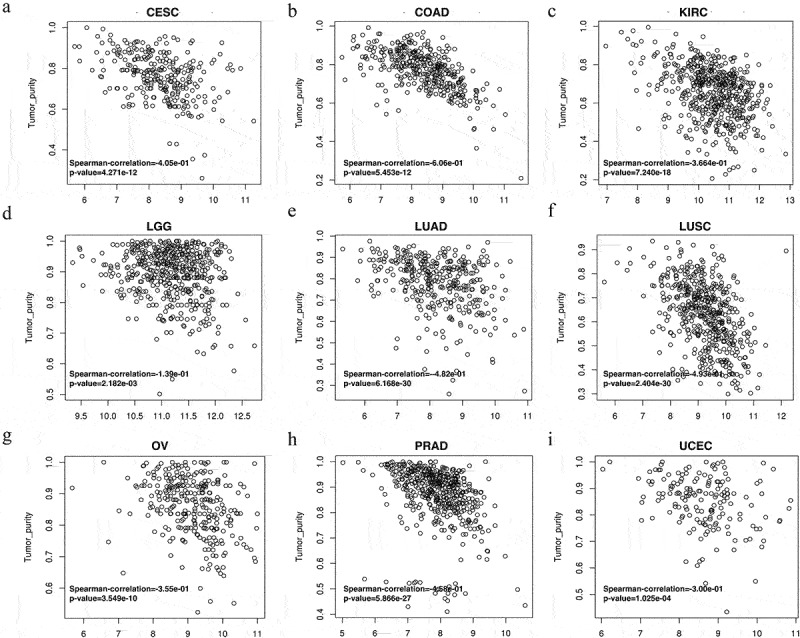
High expression of *VASH1* led to a decrease in tumor purity in human cancer. *VASH1* inversely affected tumor purity in CESC (a), COAD (b), KIRC (c), LGG (d), LUAD (e), LUSC (f), OV (g), PRAD (h), and UCEC (i). CESC, cervical squamous cell carcinoma and endocervical adenocarcinoma; COAD, colon adenocarcinoma; KIRC, kidney renal clear cell carcinoma; LGG, brain lower grade glioma; LUAD, lung adenocarcinoma; LUSC, lung squamous cell carcinoma; OV, ovarian serous cystadenocarcinoma; PRAD, prostate adenocarcinoma; UCEC, uterine corpus endometrial carcinoma. All *p* < .05.

The LinkedOmics website was used to study the association between *VASH1* expression and tumor purity. The results indicated that high expression of *VASH1* led to a decrease in tumor purity in CESC (*Spearman-Correlation*= −0.405), COAD (*Spearman-Correlation*= −0.606), KIRC (*Spearman-Correlation*= −0.366), LGG (*Spearman-Correlation*= −0.139), LUAD (*Spearman-Correlation*= −0.482), LUSC (*Spearman-Correlation*= −0.493), OV (*Spearman-Correlation*= −0.355), PRAD (*Spearman-Correlation*= −0.458), and UCEC (*Spearman-Correlation*= −0.300), which verified that *VASH1* was inversely related to tumor purity (Figure 4).

### VASH1 *impacted immune cell infiltration*

**Figure 5.**
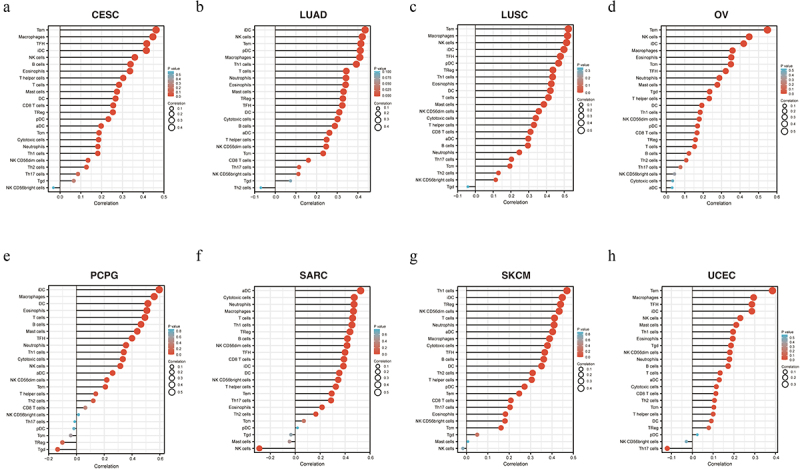
*VASH1* was associated with immune cell infiltration in numerous kinds of tumors, including CESC (a), LUAD (b), LUSC (c), OV (d), PCPG (e), SARC (f), SKCM (g), and UCEC (h). CESC, cervical squamous cell carcinoma and endocervical adenocarcinoma; LUAD, lung adenocarcinoma; LUSC, lung squamous cell carcinoma; OV, ovarian serous cystadenocarcinoma; PCPG, pheochromocytoma and paraganglioma; SARC, sarcoma; SKCM, skin cutaneous melanoma; UCEC, uterine corpus endometrial carcinoma.

In recent years, tumor immunocyte infiltration has gradually become a research focus, and tumor immunotherapy has achieved high performance. *VASH1* was relevant to immune cell infiltration in numerous types of tumors. We defined a strong correlation coefficient greater than 0.4 as a strong correlation. As shown in Figure 5, *VASH1* expression was positively correlated with Tem, macrophages, TFH, and iDC in CESC; iDC, NK cells, Tem, pDC, and macrophages in LUAD; Tem, macrophages, NK cells, iDC, TFH, and pDC in LUSC; Tem, NK cells, and iDC in OV; iDC, macrophages, DC, eosinophils, T cells, B cells, and mast cells in PCPG; aDC, cytotoxic cells, neutrophils, macrophages, T cells, Th1 cells, Treg, and B cells in SARC; Th1 cells, iDC, Tregs, NK CD56dim cells, T cells, and neutrophils in SKCM; and Tem in UCEC. By integrating the types of immune cells infiltrated in various tumors, it was found that *VASH1* mainly affected the infiltration degree of iDCs, Tems, macrophages, and NK cells.

### *The effect of* VASH1 *expression on drug therapy responsiveness in ovarian cancer*

**Figure 6.**
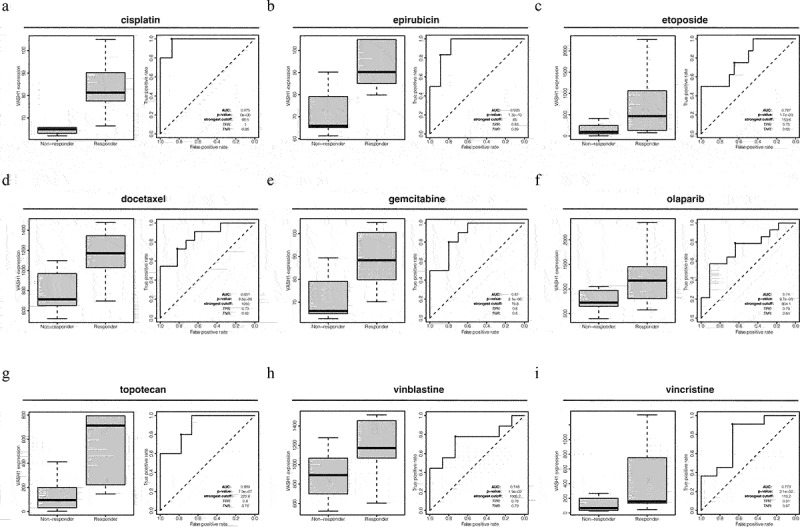
High *VASH1* expression resulted in a higher sensitivity to multiple drugs in ovarian cancer.

After comprehensively understanding the role of *VASH1* across cancers, we focused on its function in ovarian cancer. According to ROC plotter database analysis, patients with high *VASH1* expression were more sensitive to multiple first- and second-line chemotherapy drugs for ovarian cancer. *VASH1* could serve as a highly reliable detection biomarker for estimating the efficacy of chemotherapy, including cisplatin (AUC = 0.975, *p* = 0e + 00), epirubicin (AUC = 0.926, *p* = 1.3e-10), etoposide (AUC = 0.787, *p* = 1.7e-03), docetaxel (AUC = 0.851, *p* = 8.8e-06), gemcitabine (AUC = 0.870, *p* = 2.1e-06), olaparib (AUC = 0.740, *p* = 9.7e-03), topotecan (AUC = 0.889, *p* = 7.9e-07), vinblastine (AUC = 0.748, *p* = 1.9e-02), and vincristine (AUC = 0.773, *p* = 2.1e-02) (Figure 6).

### GO *and* KEGG *enrichment analysis of* VASH1 *in ovarian cancer*

**Figure 7.**
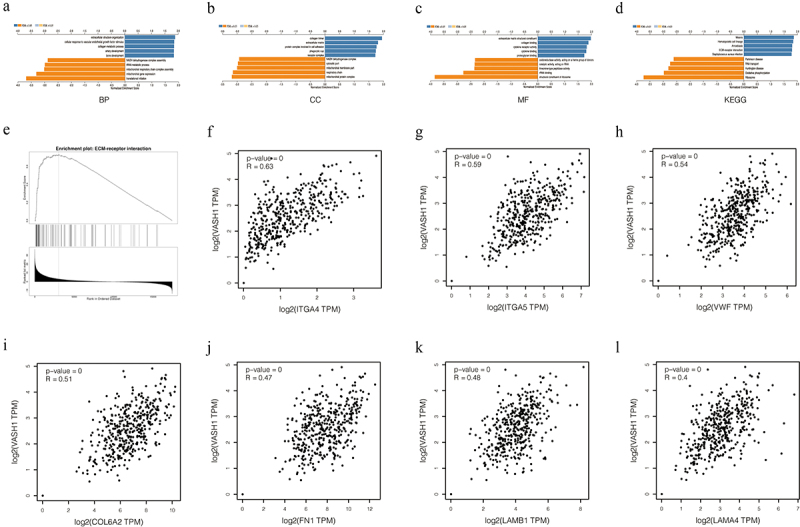
*VASH1* plays important biological functions in ovarian cancer. BP (a), CC (b), MF (c), KEGG (d), ECM-receptor interaction (e). The correlation between *VASH1* and coexpressed genes (f-l).

We identified differentially expressed genes correlated with *VASH1* in ovarian cancer and concentrated on the potential biological functions of these genes. GO function enrichment analysis showed that functions focused on translational initiation, mitochondrial gene expression, and extracellular structure organization for BP; mitochondrial protein complex, respiratory chain, and collagen trimer for CC; structural constituent of ribosome; rRNA binding; and extracellular matrix structural constituent of MF. KEGG enrichment analysis revealed that the genes were mainly involved with the ribosome, oxidative phosphorylation, and malaria. We also found that genes related to extracellular matrix receptor interaction were coexpressed with *VASH1*, including *ITGA4* (*Spearman-correlation* = 0.63, *p* = 0), *ITGA5* (*Spearman-correlation* = 0.59, *p* = 0), *VWF* (*Spearman-correlation* = 0.54, *p* = 0), *COL6A2* (*Spearman-correlation* = 0.51, *p* = 0), *FN1* (*Spearman-correlation* = 0.47, *p* = 0), *LAMB1* (*Spearman-correlation* = 0.48, *p* = 0), and *LAMA4* (*Spearman-correlation* = 0.4, *p* = 0) (Figure 7).

### VASH1 *inhibited the malignant phenotype of ovarian cancer cells*

**Figure 8.**
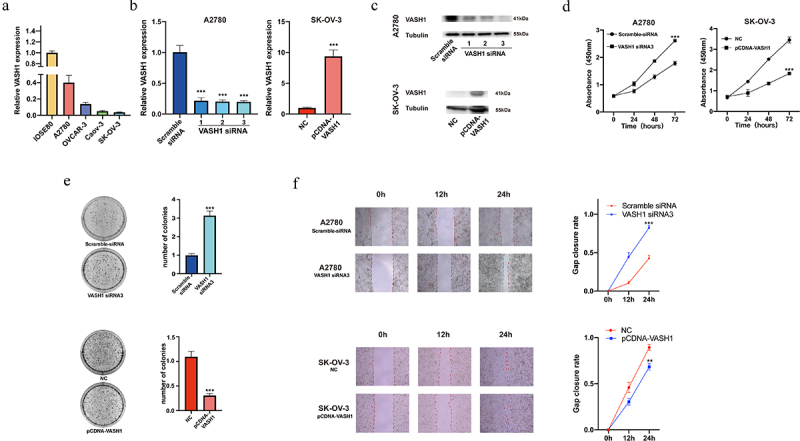
*VASH1* could restrain the malignant phenotype of ovarian cancer cells. *VASH1* expression was compared among ovarian cancer cell lines and normal ovarian epithelial cells (a). The level of *VASH1* expression was determined after transfection (b, c), proliferation assay (d), colony formation (e), and wounding healing assay (f) with *VASH1* overexpression and knockdown.

To further explore the specific role of *VASH1* in the development of ovarian cancer, we examined the expression of *VASH1* in normal ovarian epithelial cells (IOSE80) and ovarian cancer cells (A2780, OVCAR-3, Caov-3, and SKOV3). Compared with IOSE80 cells, the expression of *VASH1* was decreased in cancer cells (Figure 8a). We then constructed *VASH1* overexpression and knockdown cell lines and verified their transfection efficiency by qRT‒PCR and western blotting (Figure 8b, c). The reduction in *VASH1* expression significantly enhanced proliferation and colony formation, and *VASH1* overexpression inhibited cell proliferation (Figure 8d, e). Using the wounding healing assay, we examined the effect of *VASH1* on cell migration ability. Compared to the control group, remarkable wound healing was observed at 12 and 24 h after *VASH1* expression was downregulated. Conversely, overexpression of *VASH1* inhibited wound healing ability (Figure 8f).

## Discussion

*VASH1* has been shown to be related to the relapse and progression of various tumors by inhibiting angiogenesis.^19–21^ This has become the focus of tumor research. However, the molecular mechanism of *VASH1* has not yet been completely clarified.

Our results showed that the expression of *VASH1* in various tumors was significantly different from that in normal tissues. However, *VASH1* was consistently associated with better OS and RFS in pancancer patients. Several studies have confirmed that the expression level of *VASH1* is related to the prognosis of many solid tumors. Zhao et al.^22^ showed that high expression of *VASH1* was associated with a more satisfactory prognosis of renal cell carcinoma by inhibiting tumor angiogenesis.^23^ *VASH1* overexpression represses tumor growth and promotes apoptosis by inhibiting the G0/G1 cell cycle. At the same time, an increase in *VASH1* enhances tumor sensitivity to chemotherapy.^24^ In colon cancer, overexpression of *VASH1* remarkably reduces growth, movement, and clonogenic capacity.^25^ In ovarian cancer, *VASH1* inhibits the expression of *IGF-1* and inhibits angiogenesis.^17^ This further suggests that *VASH1* may act as an antitumor agent through diverse mechanisms. According to the ROC curve results, *VASH1* is also of high value in the prediction and diagnosis of general cancer, with high sensitivity and a low false-positive rate.

Tumor tissue is a complex mixture, including immune cells, interstitial cells, stromal cells, and other nontumor cells in tumor tissue, which commonly affect patient outcomes.^26,27^ The proportion of tumor cells is known as tumor purity.^28^ Our data showed that the expression of *VASH1* inversely affected tumor purity, which meant that we could reduce tumor purity and regulate the infiltration of immune cells in the TME by increasing *VASH1* expression. In recent years, immunotherapy has become an effective method for treating malignant tumors. Generally, chemotherapy, endocrine therapy, and targeted therapy target only tumor cells. Unlike previous biological therapies, it is a new way of regulating or enhancing the immune response of the body to tumor cells by using appropriate methods to achieve a therapeutic effect. Immunotherapy can kill tumors by strengthening the autoimmune system. Therefore, immunotherapy has become a new breakthrough in the domain of tumor therapy after surgery, chemotherapy, radiotherapy, endocrine therapy, and targeted treatment.^29,30^ We found that *VASH1* was mainly associated with iDC, macrophage, and NK cell infiltration. This mixture is considered to play a vital role in tumor growth, disease progression, and drug resistance. It has been reported that dendritic cells (DCs) account for a small part of the tumor microenvironment and are an important antitumor component with the ability to promote T-cell immunity and immunotherapeutic response.^31^ DCs play a central role in regulating the balance between CD8^+^ T-cell immunity and tumor antigen tolerance. Cross-initiation is the process by which DCs activate CD8^+^ T cells through cross-presentation of foreign antigens and plays a key role in the generation of antitumor CD8^+^ T-cell immunity.^32^ NK cells can control tumor growth by directly interacting with cancer cells and other immune cells. The presence of NK cells in the tumor microenvironment may lead to a good prognosis. The infiltration of NK cells and CD8^+^ T cells in the TME of patients with colorectal cancer was positively correlated with prolonged survival. In gastric and esophageal cancers, the proportion of infiltrating CD56dim NK cells gradually decreases with disease progression.^33^

Ovarian cancer is a gynecological malignant tumor with the highest mortality rate. Tumor cytoreductive surgery is the main treatment, followed by adjuvant chemotherapy.^34^ The 5-year survival rate of women diagnosed with high-grade serous ovarian cancer is between 35% and 40% because 15% − 25% of patients have primary treatment resistance, while most of the remaining women develop chemotherapeutic resistance.^35^ Hence, there is an urgent need to improve the sensitivity of ovarian cancer patients to therapeutic drugs. Studies have confirmed that VASH1 could affect drug sensitivity through different pathways. Shuji Mikami et al. found that the VASH1 density in metastatic clear-cell renal cell carcinoma treated with sunitinib was significantly higher than that in untreated ones, indicating that VASH1 may be related to endothelial cell resistance to sunitinib treatment.^36^ In osteosarcoma cells, it was identified that VASH1 was able to inhibit adriamycin resistance through regulation of the AKT signal pathway.^37^ In addition, miR‐335‐5p promotes insulin resistance by activating the TGF‐β pathway and inhibiting VASH1 expression.^38^ In our study, it was found that in this study increased expression of VASH1 could improve the responsiveness of ovarian cancer to many drugs, including first- and second-line chemotherapy drugs and PARP inhibitors commonly used in the clinic. This suggests that increasing the expression level of VASH1 may reverse drug resistance in patients. In this study, GSEA outcomes also showed that the expression of VASH1 was positively correlated with the ECM receptor interaction pathway in ovarian cancer, including genes related to integrin, collagen, fibronectin, and laminins. ECM receptors are the main regulatory pathways that control various cellular processes, including survival, proliferation, migration, invasion, DNA damage, signal transduction, and repair.^39^ Downregulation of ECM composition and structure is related to carcinogenesis and cancer progression.^40,41^ Therefore, we suspect that the upregulation of VASH1 is positively correlated with the ECM receptor to inhibit ovarian cancer metastasis and that VASH1 may be a possible therapeutic target. We further verified the bioinformatic analysis results of the VASH1 antitumor effect through in vitro experiments. Overexpression of VASH1 reduces cell proliferation, cloning, and migration in ovarian cancer cell lines.

## Conclusions

This study revealed the antitumor effect of *VASH1* across cancers. *VASH1* is a candidate prognostic and diagnostic marker for many types of cancers and can be used to evaluate the infiltration of immune cells into malignant tissues. Thus, *VASH1* may contribute to the comprehensive prevention and treatment of ovarian cancer as a potential therapeutic target.

## Abbreviations

VASH1: Vasohibin-1
OS: overall survival
RFS: recurrence free survival
TME: tumor microenvironment
GEPIA: Gene Expression Profiling Interactive Analysis
TCGA: The cancer genome atlas
GO: gene ontology
KEGG: Kyoto encyclopedia of genes and genomes
ACC: adrenocortical carcinoma
LUAD: lung adenocarcinoma
LUSC: lung squamous cell carcinoma
OV: ovarian serous cystadenocarcinoma
UCEC: uterine corpus endometrial carcinoma
CHOL: cholangiocarcinoma
ESCA: esophageal carcinoma
KIRC: kidney renal clear cell carcinoma
KIRP: kidney renal papillary cell carcinoma
LIHC: liver hepatocellular carcinoma
SKCM: skin cutaneous melanoma
THCA: thyroid carcinoma
HNCS: head and neck squamous cell carcinoma
PAAD: pancreatic adenocarcinoma
READ: rectal adenocarcinoma
STAD: stomach adenocarcinoma
CESC: endocervical adenocarcinoma
COAD: colon adenocarcinoma
LGG: brain lower grade glioma
PCPG: pheochromocytoma and paraganglioma
SARC: sarcoma
Tem: effector memory T Cell
TFH: follicular helper T cell
DC: dendritic cell
iDC: interdigitating dendritic cell
NK: natural killer cell
pDC: plasmacytoid dendritic cell
Adc: active dendritic cell
BP: biological process
CC: cell component
MF: molecular function
ITGA4: integrin, alpha 4
ITGA5: integrin, alpha 5
VWF: von Willebrand factor
COL6A2: collagen, type VI, alpha 2
FN1: fibronectin 1
LAMB1: laminin, beta 1
LAMA4: laminin, alpha 4
IGF-1: insulin-like growth factor 1
PARP: poly ADP-ribose polymerase
GSEA: Gene Set EnrichmentAnalysis
ECM: extracellular matrix.
